# Chicago Pathological Society

**Published:** 1883-09

**Authors:** J. H. Tebbetts

**Affiliations:** Secretary


### ARTICLE IX.

CHICAGO PATHOLOGICAL SOCIETY.

Regular meeting, June 11, 1883.

The Society was called to order by the President, Dr. Angear.

The minutes were read and approved.

Drs. Isabell R. Copp and C. A. Sanders were elected to membership.

The following named gentlemen were proposed for membership: Drs. R. N. Hall, J. E. Harper and E. E. Holroyd, by Drs. Angear and Tebbets.

The Society then listened to the reading of a paper by Dr. E. P. Murdock, entitled, "The Immediate Operation for Repair of Lacerated Cervix."

The author stated that reproduction is a pathological process, although a proposition not in accordance with the views of the most eminent teachers of physiology; he would not limit the term pathology to conditions wholly unnatural, as death is a natural process, and all diseases are the result of natural causes, but to designate processes and conditions which sow the seeds of disease and death, or establish to train of symptoms which produce these causes of death.

The author then enumerated as pathological processes, the destruction of the hymen in coition, nausea of pregnancy, the disordered nutrition during gestation, the œdema of the limbs, the laceration of the cervix and perinæum, and the separation of the placenta from the uterine walls. In the vegetable kingdom, all annual plants die as a result of efforts at reproduction, and perennial plants, long lived when sterile, become weak and succumb to the exhausting influence of reproduction.

In the animal kingdom, many varieties die soon after bringing forth offspring.

In women, quoting from Prof. Byford, the genital apparatus is in a constant state of predisposition to disease.

The author considered a pregnant women as a patient to be kept under observation for a long time before and after delivery.

The text-books devote but little space to the immediate operation, but describe Emmet's operation at some length.

Dr. Pallen early called attention to the subject in 1866, by reports of cases, and again in 1874, also, Dr. Nelson, in 1877, had some successful cases.

Many cases pass unrecognized, owing to the soft condition of the parts, and the amount of blood covering them. To render diagnosis easier, one should note the nature of the hæmorrhage, which is arterial ; sometimes a tear is detected as the head is passing through the os.

The reliable method adopted by careful examiners is to draw down the uterus with Vulsellum forceps, and expose to vision. The doctor then reported three cases as operated upon, two of which were highly successful, one being unsuccessful ; and in conclusion, remarked that the injury could and should be recognized at the time or parturition, and that the importance of the operation cannot be overestimated, because—

1st. All operations for repair of injury should be done at the earliest possible moment, to secure repair by first intention, prevent deformity and prevent sepsis.

2d. The patient is saved all trouble and annoyance of preparatory treatment for a secondary operation.

3d. It gives the patient the best chance to escape septicæmia, subinvolution, and other complications which follow cervical laceration.

4th. It saves much time, great expense, and avoids deformity which would always remain unrepaired by plastic surgery.

The paper being before the society for discussion, Dr. Lyman expressed much interest in listening to its reading, but thought that experiment alone would decide the value of the operation. He differed from the writer, in not considering pregnancy a pathological condition. Many women are ill at time of delivery, but the majority of cases present phenomena as natural as any in nature. If diseased, we should look for the cause, and consider it coincidental only; nausea, many hold, is only a normal condition of this state, protecting against miscarriage. In many cases, where an abnormal tendency to miscarriage existed, nausea should be induced, quoting Dr. Bedford. Artificial conditions produce accidental complications. Lacerations may often be left to nature. There is a remarkable tendency to recovery in lacerations of the perineum; one month after labor, on examination, one is astonished to note the great repair, without operative interference, although one had been tempted to operate.

Position and rest will go far toward promoting union. There are failures in many cases of operation. He considered the condition of patient at time of parturition very bad for operation, and that it was unwise, unless compelled, to add to the danger of septic poisoning. In rare cases only, after involution was completed, should the knife be used. Gentle topical treatment was preferable.

Dr. C. J. Lewis would not regard gestation as a pathological condition, but rather a hypertrophic one; he would not operate immediately after labor, for reason of adding surface to already large one for sepsis to occur. In wounds of the perineum would always apply deep, well adjusted sutures. The difficulty often found in obtaining consent of patient for a secondary operation would be an inducement to perform the immediate operation.

Dr. Bishop thought lacerations might be prevented by more careful finger dilatation.

Dr. Valin considered gestation a pathological process in the human female; in operation for laceration of the perineum had failed with silk sutures, but with the silver would be sanguine of success.

Dr. Lagorio had two cases of laceration of cervix after using forceps; thought many physicians are too prone to use forceps, doing much damage, and causing many lacerations; did not understand how union by first intention could occur on a surface in a pathological condition. The operation was at all events a serious one—the patient being already fatigued.

Dr. Van Buren would demand facts to prove that gestation is a pathological condition in the majority of cases, though in the vegetable kingdom the fact might be true where the soil was sterile. He would hardly sit still and wait for nature to repair a laceration. There were some advantages in the immediate operation, *i. e.*, the relaxation of the uterus, allowing that organ to be drawn down and cleansed thoroughly; also in the conclusion of operation at one and same time with labor; its disadvantages seemed to be exhaustion and the lochia, unfavorable conditions to treat a wound; would be so considered in wounds elsewhere. Experiment was to be continued, and for this he waited.

Dr. Murdock, in closing the discussion, felt pleased with the thorough criticism of the members, but differed in a few points. Would not the torn surfaces be best protected from the lochia when well coapted? The careful operator will not operate on an exhausted woman. That the accoucheur of necessity has judgment, is pre-supposed. Probably not one case in twenty but what will bear the operation. Many are waiting for further light on the subject; from whence will it reach us, unless some one operates?

In every case of those now operating they report favorably, although many authorities are not now in favor of operation for repair of the perineum, while still favoring the operation upon the cervix.

So far as can be ascertained, success is the result in 65 per cent. of cases. Our main reason for operating is to prevent septicæmia. The operation originated in attempting to check arterial hæmorrhage.

Dr. Lyman moved that a vote of thanks of the society be tendered Dr. Murdock, and that an abstract of the paper be furnished for publication. Carried.

Dr. Angear, as chairman of the committee on rooms for the society, reported that the College of Physicians and Surgeons had offered accommodations, including cases in the college museum, for storage of pathological specimens, hence it seemed best to the committee to call the meeting at this place, to ascertain the wishes of the society, and try the location, whether sufficiently central, etc.

Dr. Lyman moved that the society accept the offer of the college. Carried unanimously. Dr. Lyman also moved that the officers of the society interest the staff of the County Hospital in securing interesting specimens, reports of cases, etc. Carried. Dr. E. P. Murdock was unanimously elected curator of the society.

Among the members present were Drs. Angear, Bennett, Bishop, Bucher, Dobbin, Hanson, Lagorio, Lewis, Lyman, Leyon, Murdock, Newton, Patton, Sage, Tagert, Tebbetts, Valin; also four visitors.

The society, on motion, adjourned.

J. H. TEBBETTS, *Secretary.*

---

### ARTICLE X.

### CHICAGO PATHOLOGICAL SOCIETY.

July 9. Regular meeting. The meeting was called to order by the President, Dr. Angear. The minutes were read and approved.

The following named gentlemen were elected to membership:

